# Initiation of lactation and the provision of human milk to preterm infants in German neonatal intensive care units from the mothers’ perspective

**DOI:** 10.1186/s12884-022-04468-7

**Published:** 2022-02-25

**Authors:** N. Scholten, L. Mause, D. Horenkamp-Sonntag, M. Klein, T. Dresbach

**Affiliations:** 1 Institute of Medical Sociology Health Services Research and Rehabilitation Science, Faculty of Human Sciences and Faculty of Medicine, University of Cologne, Cologne, Germany; 2 Techniker Krankenkasse, Healthcare Management, Hamburg, Germany; 3 DAK Gesundheit, Hamburg, Germany; 4 Department of Neonatology and Pediatric Intensive Care Medicine, University Hospital Bonn, Bonn, Germany

**Keywords:** Human milk, Lactation, Preterm, Very low birth weight, Neonatal intensive care unit

## Abstract

**Background:**

If infants with a very low birth weight (VLBW) are to be fed exclusively with human milk, it is essential to focus on lactation initiation. The aim of the study is to learn more about the current state of lactation initiation and human milk provision in neonatal intensive care units in Germany from the mothers' perspective.

**Methods:**

Written surveys were conducted with mothers of VLBW infants to learn more about the timing of initiation of lactation, pumping frequency during the first three days postpartum and feeding of the preterm infant during hospitalisation.

**Results:**

The data of 437 mothers (response rate: 44.7%) were included in the analyses. Of these, only 7.8% stated that they had initiated lactation immediately after delivery and 38.2% within 6 h. In terms of pumping frequency, 50.1% pumped 7–9 times a day within the first 3 days postpartum; 60.9% reported that their infant received formula feedings during the hospital stay.

**Conclusion:**

Overall, deficits were still evident with regard to the initiation of lactation in mothers of VLBW infants in Germany, resulting in a large proportion of VLBW infants receiving formula in the hospital.

**Trial registration:**

German Clinical Trial Register: DRKS00017755.

## Background

It is undisputed that mother’s milk (MOM) is the best nutrition for every newborn. For more than 15 years, the WHO has recommended exclusive feeding with human milk from the first day of life [1]. Optimal nutrition with human milk is particularly crucial for the further development of vulnerable newborns, such as children with very low birth weight (VLBW, < 1500 g) and extremely low birth weight (ELBW, < 1000 g) as well as newborns with, for example, complex congenital heart diseases [2–4]. Human milk has a crucial role in the prevention of necrotizing enterocolitis (NEC) [5], and MOM on late onset sepsis, retinopathy of prematurity (ROP) and bronchopulmonary dysplasia (BPD) [6, 7].

If MOM is not available, donor milk is preferable to formula for premature infants [8]. This is justified by the lower rates of NECs in preterm infants confirmed in several studies [7, 9, 10]. At the same time, the availability of donor milk does not reduce the rate of discharged infants fed with MOM [11] but, rather, increases it [12]. Thus, the application of formula is justified only when MOM and donor milk are not available [13].

If the goal is to provide MOM to premature infants and explicitly to infants with a VLBW, it is important to provide the mothers of preterm infants with structured support in terms of lactation. Early first milk expression has been shown to be particularly relevant for the successful initiation of lactation in mothers of VLBW infants. Based on older, underpowered studies the initial expression during the first hour postpartum (p.p.) is often propagated [14, 15] although recent studies assume that an initial expression within the first 181–360 min p.p. should be aimed for [16, 17]. Premature birth complicates the onset of secretory activation, which is relevant to producing a sufficient amount of milk [18]. In addition to expressing milk early, attention should be paid to regular stimulation of the breasts by expressing milk [19, 20]. Successful achievement of full lactation is best achieved by expressing milk at least six times daily [21]. If the goal is to provide MOM to as many preterm infants as possible, and explicitly to those with a VLBW, these mothers must be supported. In addition to early milk expression, continuous support, e.g. through counselling or reminders from the medical team, is useful for this purpose [19, 22].

To date, little is known about lactation initiation and current lactation promotion in German neonatal intensive care units (NICUs). In contrast to clinical trials, the focus here is on the subjective view of the mothers. The aim of this survey was, therefore, to investigate lactation initiation practice during the first 3 days p.p. and lactation support from the mother’s perspective of VLBW premature infants in Germany. The objective of the analyses was to relate these factors to the nutrition of the preterm infant (MOM, donor milk or formula) during hospitalization.

## Methods

The results were obtained from a parent survey, which constitutes a separate work package within Neo-CamCare. The focus of the parent survey was on the experiences of parents of preterm infants and their attitudes towards webcams. The experiences in relation to lactation initiation support were queried as part of the parental experiences. A survey of mothers of preterm infants weighing less than 1500 g at birth was conducted in cooperation with two statutory health insurance companies. The two health insurance companies are cooperative partners in the study. On the one hand, they take over the recruitment of the survey participants, and on the other hand, they support the transfer of results. The participation of the two health insurance companies makes it possible to address mothers of VLBW infants without self-selection bias and without focusing on individual clinics. In this way, mothers whose children were cared for in different clinics can be reached throughout Germany. However, the health insurance companies are not given any individual information about the mothers that was provided in the questionnaire (anonymous survey).

Mothers of preterm infants under 1500 g (ICD-10-GM selection criterion within the health insurance claims data: P07.00, P07.01, P07.02, P07.10, P07.11) whose children were between 6 and 18 months old at the time of the survey were invited to participate in the survey by the health insurance companies. Mothers whose child had died at the time of the survey were not contacted. In total, 1001 mothers were invited by the health insurers to participate in the written survey by sending them the written questionnaire, asking them to fill it in and return it to the Institute of Medical Sociology, Health Services Research and Rehabilitation Science in a pre-stamped envelope by post. The recruitment was carried out completely by the health insurance companies, without participation of the clinics. For this reason, an insight into the files as well as a characterization of the clinics providing care was not possible. In Germany, infants with a birth weight below 1500 g may only be cared for in level 1 and 2 perinatal centres. These represent the highest level of care and are comparable to the US-American level 3 and 4 centres.

The time frame was chosen in such a way that the time spent in the NICU could be considered with a certain temporal distance in order to prevent re-traumatization, but was recent enough to minimize possible distortions due to recall bias.

The written survey was conducted between September and December 2020. Study participants were reminded once with a combined thank-you and reminder letter. As an incentive to participate in the study, a small gift (vaccination passport cover) was enclosed, and prizes (including shopping vouchers and first aid kits for children) were raffled off among the participants in a separate lottery. Participation in the lottery was independent of participation in the study. Thus, it was possible to participate in the raffle without having returned the questionnaire, as participation in the lottery was possible via a separate postcard, keeping the survey itself anonymous. In total, 447 mothers participated in the survey, representing a response rate of 44.6%.

### Survey instrument

The survey instruments explicitly presented here on lactation support of MOM, donor milk and formula supply in German NICUs were developed by an interdisciplinary team consisting of a neonatologist and a NICU experienced nurse as well as health and social scientists, based on the current literature as well as the personal experience of the clinicians. The questionnaire was pre-tested by one mother of a premature child and the self-help group of parents of prematurely born children.

The survey questions included the time of first pumping and the frequency of pumping in the first 3 days p.p. Furthermore, support by medical staff was surveyed with regard to the time of introduction to the use of the pump, reminders to pump and the promotion of feeding of premature infants with human milk, stratified according to physicians and nursing staff. The nutrition of the premature infant during the inpatient stay was also recorded by questioning the mother; a distinction was made between MOM, donor milk and formula. For each type of nutrition, a choice could be made between exclusively, mostly, partially or not at all.

In addition to the descriptive presentation of the results, a multivariate analysis of possible influencing factors was performed. For the univariate and multivariate analyses, single dependent variables have been summarized as described below as the population size was sometimes very small. In the analyses, birth weight was dichotomized into above and below 1000 g, and regarding the time of initiation of lactation the categories “within the first 48 h” and “within the first week” were merged. The education categories were summarized into still in education/no education, completed education/technical and vocational training, and university degree. Additionally, sociodemographic data of the mothers, gestational age and multiple child status of the VLBW infant were considered. The analyses were performed with Stata 16. Excluded from the analyses were questionnaires received twice (for multiples) (*n* = 5) and the data for mothers whose children did not fall within the defined age range (*n* = 5).

## Results

The responses of a total of 437 mothers were included in the analyses. The sociodemographic data of the mothers surveyed and information on the child’s birth weight and multiple pregnancy status can be found in Table 1.

**Table 1:** Sociodemographic data of the mothers and data of the VLBW infants

Mother’s age (Mean, Min–Max, SD)	34.3 (21–53, SD: 4.5)
Highest graduation certificate achieved:	
Without a school graduation certificate	2.1% (*n* = 9)
Still in vocational training (training, internship, student)	0.5% (*n* = 2)
Completed vocational training	47.1% (*n* = 206)
University degree	49.7% (*n* = 217)
Missing	0.7% (*n* = 3)
Native language	
German	84.7% (*n* = 370)
Another native language	14.9% (*n* = 65)
Missing	0.5% (*n* = 2)
Birth weight of the child	
< 500 g	2.5% (*n* = 11)
500–999 g	38.2% (*n* = 167)
1000–1499 g	58.1% (*n* = 254)
Missing	1.1% (*n* = 5)
Multiple pregnancies	
Yes	35.5% (*n* = 155)
No	63.2% (*n* = 276)
Missing	1.4% (*n* = 6)

**Fig. 1:**
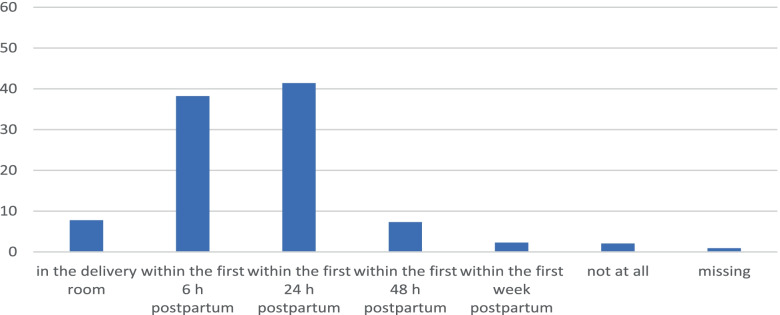
Initiation of lactation (first time pumping)

**Fig. 2:**
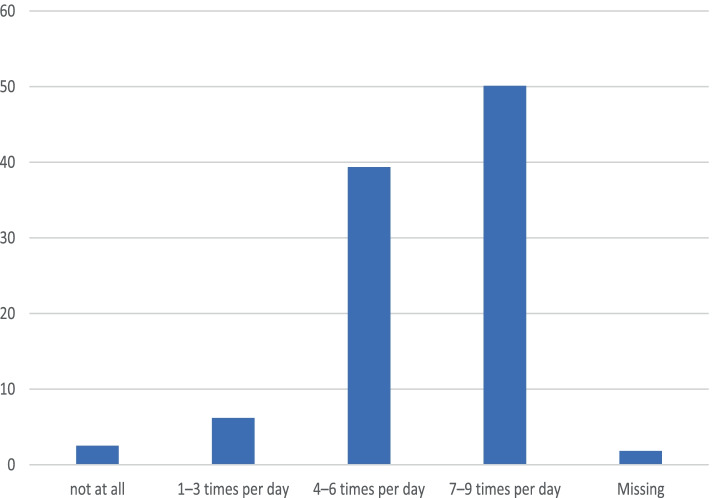
Pumping Frequency within the first 3 days postpartum

Initial pumping was reported to have been performed in the delivery room and therefore immediately after delivery by 7.8% (*n* = 34) of the questioned women. Within 6 h of delivery, 38.2% (*n* = 167) and within 24 h of delivery, 41.4% (*n* = 181) of women pumped initially. In terms of pumping frequency, it appears that the majority (50.1%, *n* = 219) of women pumped 7–9 times daily within the first 3 days. Details can be found in Fig. 1 and in Fig. 2.

In terms of reminders about regular pumping by health professionals, wide disparities were reported, with the majority of mothers surveyed (52.6%, *n* = 230) reporting never having been reminded. On the other hand, 22.0% of mothers (*n* = 96) reported having been reminded several times a day.

During hospitalization, 34.6% (*n* = 151) of the mothers who answered the question reported that the VLBW infant had been fed exclusively with MOM, 14.7% (*n* = 64) of VLBW infants received donor milk and 60.9% (*n* = 266) received formula. Details can be found in Table 2.

**Table 2:** Nutrition of the VLBW infant during hospitalization

	MOM n (%)	Donor Milk n (%)	Formula n (%)
Exclusively	151 (34.6)	1 (0.2)	20 (4.6)
Most of the time	156 (35.7)	11 (2.5)	71 (16.3)
Sometimes	114 (26.1)	52 (11.9)	175 (40.1)
Not at all	6 (1.4)	259 (59.3)	106 (24.3)
Missing	10 (2.3)	114 (26.1)	65 (14.9)

Of the infants whose mothers pumped immediately after delivery, 61.8% (*n* = 21) were fed exclusively with MOM. If milk was pumped within the first 6 h, the percentage decreased to 43.3% (*n* = 71) and if pumped within the first 24 h, to 26.5% (*n* = 48). Similar effects were seen in relation to pumping frequency. Of mothers who reported pumping 7–9 times in the first 3 days, 45.9% (*n* = 100) also reported that their infant received MOM exclusively. This figure decreased from 26.3% (*n* = 45) when pumping 4—6 times per day to 15.4% (*n* = 4) when pumping 1—3 times. The chi-squared test showed a strong significant association between the time of initial pumping and the pumping frequency within the first 3 days (*p* = 0.000). Mothers who reported not having pumped were excluded from this and the following analyses (*n* = 9). Details can be found in Table 3.

**Table 3:** Relationship between exclusive feeding with MOM and initiation time and frequency

Only MOM	Initiation of Lactation within			Total
	< 6 h	6–24 h	< 24 h	
no	53.5%	73.5%	78.6%	
	*n* = 106	*n* = 133	*n* = 33	272
yes	46.5%	26.5%	2.4%	
	*n* = 92	*n* = 48	*n* = 9	149
Total	198	181	42	421
Pearson chi-squared = 20.4, *p* = 0.000				
Only MOM	Pumping Frequency (3 days postpartum)			Total
	7–9	4–6	1–3	
no	54.1%	73.7%	84.6%	
	*n* = 118	*n* = 126	*n* = 22	266
yes	45.9%	26.3%	15.4%	
	*n* = 100	*n* = 45	*n* = 4	149
Total	218	171	26	415
Pearson chi-squared = 21.0, *p* = 0.000				

### Maternal, sociodemographic and child factors influencing the timing of initial pumping and pumping frequency

With regard to the timing of initial pumping, the multivariate models did not identify any significant influencing factors, neither on the maternal side (age, education, native language) nor on the infant side (birth weight, multiple births). By contrast, when pumping frequency was considered, the multivariate regression model (ordered logistic regression) showed a significant association of the child’s birth weight (*p* = 0.001), with mothers of infants under 1000 g pumping significantly more often. Neither sociodemographic data of the mothers (age, education, native language) nor multiple births played a significant role. The multivariate logistic regression model can be found in Table 4.

**Table 4:** Maternal and child factors associated with the time of initiation and frequency (ordinal logistic regression)

	Model: Initiation			Model: Frequency		
	Pseudo - *R*^2^ = 0.0039			Pseudo - *R*^2^ = 0.0301		
Independent variables (reference categories)	Odds Ratio	*p* > |z|	95% CI	Odds Ratio	*p* > |z|	95% CI
Birth weight (ref. < 1000 gramms)				0		
1000 – 1.500 gramms	1.019	0.921	0.706 to 1.471	01.980	0.001***	1.325 to 2.958
Multiple (ref. Yes)						
No	1.193	0.358	0.819 to 1.738	1.401	0.110	0.926 to 2.119
Education (ref. without / in training)						
Completed vocational training	0.593	0.392	0.179 to 1.962	0.358	0.093	0.108 to 1.189
University degree	0.613	0.422	0.186 to 2.021	0.355	0.089	0.108 to 1.173
Age	1.035	0.116	0.992 to 1.079	1.046	0.055	0.999 to 1.094
Native Language (ref. German)						
Other	1.044	0.874	0.612 to 1.782	1.254	0.431	0.714 to 2.204

***p ≤ 0.001

### Maternal, sociodemographic and child factors associated with the nutrition of preterm infants fed exclusively MOM

In multivariate analyses taking into account sociodemographic factors such as education and native language, it was shown that the probability that the preterm infant was fed exclusively MOM in the hospital was significantly higher in singletons than in multiples (odds ratio: 2.39, *p* = 0.001). Compared with preterm infants whose mothers pumped 7–9 times daily, preterm infants whose mothers pumped 4–6 times daily had significantly lower odds (OR 0.419, *p* = 0.000) of being fed exclusively with MOM. Pumping within the first 6 h is significantly related with the odds of the infant being fed exclusively on MOM (vs. < 24 h (OR 0.450, *p* = 0.001) vs. > 24 h (OR 0.353, *p* = 0.021)). With regard to the infant factors, there is a significant association in relation to the weight of the child, whereby children with a birth weight between 1000 g and 1499 g have a lower chance of being fed exclusively with MOM (OR 0.635, *p* = 0.049) compared with infants weighing under 1000 g. The mother’s education, native language and age did not significantly correlate with the dependant variable in the multivariate model. Overall, 11.4% of the variance can be explained by the model (Table 5).

**Table 5:** Factors associated with exclusive feeding with MOM (multivariate regression)

	Pseudo-R^2^ = 0.1139		
Independent variables (reference categories)	Odds Ratio	p >|z|	95% CI
Birth weight (ref. < 1000 g) 1000 g–1499 g	0.635	0.049*	0.404–0.999
Multiple (ref. Yes) No	2.394	0.001**	1.464–3.915
Lactation initiation (ref. within 6 h postpartum)			
6–24 h	0.450	0.001**	0.282–0.719
> 24 h	0.353	0.021*	0.145–0.856
Frequency (ref. 7–9 times)			
4–6 times	0.419	0.000***	0.260–0.677
1–3 times	0.205	0.008**	0.063–0.666
Education (ref. without/in training)			
Completed vocational training	0.274	0.063	0.070–1.073
University degree	0.404	0.190	0.104–1.568
Age	0.973	0.317	0.922–1.027
Native language (ref. German) Other	1.506	0.203	0.802–2.826
_cons	1.730	0.632	0.184–16.307

Mathematical rounding to three decimal places

*p ≤ 0.05

**p ≤ 0.01

***p ≤ 0.001

*Note:* _cons estimates baseline odds

## Discussion

The aim of the study was to learn more about the initiation of lactation and the supply of MOM and/or donor milk from the perspective of mothers of preterm infants. Current evidence suggests that the first initiation of lactation should occur within the first 6 h p.p. [16]. This is in contrast to previous studies, which emphasize the positive effect of the first initiation of lactation within the first hour p.p. [14, 23, 24]. In our survey, 7.8% of the mothers surveyed stated that they had pumped immediately after delivery, 38.2% pumped within the first 6 h and 41.4% stated that they had only pumped within the first 24 h p.p.

Despite the available evidence for emptying of the breast within the first 6 h [14, 16, 23, 25], many mothers seem to initiate lactation too late. With respect to the maternal and child factors of influence, which we have collected here, we see no significant factors associated with the timing of initiation, suggesting that the timing of initiation is primarily influenced by organizational factors within the treating hospitals [26, 27]. The knowledge of the professionals involved, such as neonatologists, nurses, midwives and gynaecologists, must be addressed, especially concerning the importance of early initiation of lactation for long-term breastfeeding success [28]. In addition to knowledge, it is known that there are other factors that influence the supply of children with human milk in the sense of a culture of family-centred care within the NICU [29]. In addition to the timing of initiation described in the literature, the frequency of pumping is used to increase the quantity of milk expressed. It has been shown that pumping intervals of 10 times [24, 30, 31] or at least 6 times in 24 h [21] should be aimed for in order to establish full lactation, where possible. In addition to early initiation, there is also a need for optimization with regard to pumping frequency, although organizational factors should also be considered here. The fact that so many mothers state that they were not reminded to pump may be a sign of the lack of focus on the human milk supply in German NICUs. The fact that mothers of children with a birth weight of less than 1000 g pumped significantly more frequently than mothers of children weighing more than 1000 g may indicate that the NICUs providing care in these cases provided more education and information. Contrary to what is described in the literature [32], we did not observe any significant effects with regard to other sociodemographic factors, such as education or age.

### Limitations

Overall, a relatively high response rate in comparison with other study populations (44.7%) was achieved with this procedure and the request to take part in the survey, which was sent by the cooperating health insurance funds. Even though health insurance is compulsory in Germany, it is not impossible that the insured clientele of the cooperating funds may not be representative, although the cooperating funds are among the largest in Germany, covering almost 20 percent of the population. In contrast to the German population average, we observed a larger proportion of mothers with higher education among our study population. This may indicate a possible selection bias in the sense that willingness to participate in the survey was positively associated with a higher education level. Even if the response rate is in line with those reported in the literature, selection bias based on the response rate cannot be ruled out. At the same time, however, the data were collected nationwide and thus cover the experiences of mothers from different NICUs and therefore apply to most of the German NICUs. Since recruitment did not take place via the NICUs, a selection bias in this respect can be ruled out, but at the same time it was not possible to collect objective information on the hospitals providing care, even though this would certainly have provided further relevant information. The data collected are based on mothers' self-reports and may be skewed by selection and confirmation bias, as in terms of later justification, memory could be biased. Regarding memory, only the data of mothers whose children were 18 months old or younger at the time of the survey were included in the study. Basically, there seems to be no systematic recall bias with regard to the pumping frequency or the time of the first pumping (no significant correlation between these two factors and the age of the child at the time of the questionnaire). The relatively late timing of 6–18 months after delivery was chosen to protect mothers from possible re-traumatisation and to avoid inviting mothers whose children were still in critical condition to participate in the survey. The question of how much mothers know about how their child is fed can be critically discussed. If donor milk is given, it is necessary in Germany to obtain the consent of the parents. It can therefore be assumed that mothers know when their child has received donor milk. However, the high number of missing values, especially for nutrition with donor milk, indicates that many mothers in Germany are still unaware of the concept of donor milk. Based on the Thomas theorem, according to which situations that are considered real have real consequences, we see the relevance of recording how mothers assess that their child has been fed [33]. The survey asked about the first pumping after delivery, with a distinction being made between the time immediately after delivery or within the first 6 h, 24 h, 48 h or first week p.p. We did not ask explicitly about the manual expression of the breast. In the future, manual milk expression could also be recorded here.

Despite the highly significant associations, the regression model presented here explains only a small proportion of the variance (11.4%). This can be attributed to the fact that other relevant factors (such as mode of birth, maternal comorbidities or organizational factors on the NICU level) could not be sufficiently taken into account. We plan to address this in future surveys. Nevertheless, these data provide initial insights into the first promotion of lactation from the mothers' perspective and are thus starting points for further questions with data beyond this.

## Conclusion

The significant association of early initiation of lactation and frequent pumping with exclusive feeding of preterm infants with MOM is evident in the survey data. If the aim is to feed children exclusively with MOM in order to prevent possible complications and improve health outcomes, these points (initiation and pumping frequency) must be addressed. The fact that, despite the highly significant results for these two factors, the model can explain relatively little of the variance shows that other factors not investigated here are still relevant to the nutritional status achieved. Among other things, the organizational climate in relation to a breastfeeding-friendly environment should be mentioned here [34, 35]. In addition to the breastfeeding-friendly climate, another point that can support the supply of MOM in the long term is the availability of donor milk. Here too, we see opportunities for more children to be supplied with donor milk (in our sample: 14.7%), at least temporarily, with the aim of being supplied with MOM in the medium term [12]. According to Klotz et al., only limited donor milk banks have been implemented so far, although in these the supply of MOM has been improved as a result [36].

## Data Availability

The datasets used and/or analysed during the current study are available from the corresponding author on reasonable request.

## References

[1] World Health Organization (2017). Guideline.

[2] Cognata A, Kataria-Hale J, Griffiths P, Maskatia S, Rios D, O'Donnell A (2019). Human milk use in the preoperative period is associated with a lower risk for necrotizing enterocolitis in neonates with complex congenital heart disease. J Pediatr.

[3] Martin CR, Ling P-R, Blackburn GL (2016). Review of infant feeding: key features of breast milk and infant formula. Nutrients.

[4] Dutta S, Singh B, Chessell L, Wilson J, Janes M, McDonald K (2015). Guidelines for feeding very low birth weight infants. Nutrients.

[5] Patel AL, Kim JH (2018). Human milk and necrotizing enterocolitis. Semin Pediatr Surg.

[6] Miller J, Tonkin E, Damarell RA, McPhee AJ, Suganuma M, Suganuma H (2018). A systematic review and meta-analysis of human milk feeding and morbidity in very low birth weight infants. Nutrients.

[7] Taylor SN (2019). Solely human milk diets for preterm infants. Semin Perinatol.

[8] Weaver G, Bertino E, Gebauer C, Grovslien A, Mileusnic-Milenovic R, Arslanoglu S (2019). Recommendations for the establishment and operation of human milk banks in Europe: a consensus statement from the European Milk Bank Association (EMBA). Front Pediatr.

[9] Altobelli E, Angeletti PM, Verrotti A, Petrocelli R (2020). The impact of human milk on necrotizing enterocolitis: a systematic review and meta-analysis. Nutrients.

[10] Quigley M, Embleton ND, McGuire W (2019). Formula versus donor breast milk for feeding preterm or low birth weight infants. Cochrane Database Syst Rev.

[11] Cañizo Vázquez D, Salas García S, IzquierdoRenau M, Iglesias-Platas I (2019). Availability of donor milk for very preterm infants decreased the risk of necrotizing enterocolitis without adversely impacting growth or rates of breastfeeding. Nutrients.

[12] Kantorowska A, Wei JC, Cohen RS, Lawrence RA, Gould JB, Lee HC (2016). Impact of donor milk availability on breast milk use and necrotizing enterocolitis rates. Pediatrics.

[13] Hay WW, Hendrickson KC (2017). Preterm formula use in the preterm very low birth weight infant. Semin Fetal Neonatal Med.

[14] Parker LA, Sullivan S, Krueger C, Mueller M (2015). Association of timing of initiation of breastmilk expression on milk volume and timing of lactogenesis stage II among mothers of very low-birth-weight infants. Breastfeed Med.

[15] Murray EK, Ricketts S, Dellaport J (2007). Hospital practices that increase breastfeeding duration: results from a population-based study. Birth.

[16] Parker LA, Sullivan S, Kruger C, Mueller M (2020). Timing of milk expression following delivery in mothers delivering preterm very low birth weight infants: a randomized trial. J Perinatol.

[17] Hoban R, Bowker RM, Gross ME, Patel AL (2021). Maternal production of milk for infants in the neonatal intensive care unit. Semin Perinatol.

[18] Cregan MD, de Mello TR, Kershaw D, McDougall K, Hartmann PE (2002). Initiation of lactation in women after preterm delivery. Acta Obstet Gynecol Scand.

[19] Nyqvist KH, Häggkvist A-P, Hansen MN, Kylberg E, Frandsen AL, Maastrup R (2013). Expansion of the baby-friendly hospital initiative ten steps to successful breastfeeding into neonatal intensive care: expert group recommendations. J Hum Lact.

[20] Hill PD, Aldag JC, Chatterton RT, Zinaman M (2005). Primary and secondary mediators' influence on milk output in lactating mothers of preterm and term infants. J Hum Lact.

[21] Ru X, Huang X, Feng Q (2020). Successful full lactation achieved by mothers of preterm infants using exclusive pumping. Front Pediatr.

[22] Miracle DJ, Fredland V (2007). Provider encouragement of breastfeeding: efficacy and ethics. J Midwifery Womens Health.

[23] Parker LA, Sullivan S, Krueger C, Kelechi T, Mueller M (2012). Effect of early breast milk expression on milk volume and timing of lactogenesis stage II among mothers of very low birth weight infants: a pilot study. J Perinatol.

[24] Becker GE, Smith HA, Cooney F (2016). Methods of milk expression for lactating women. Cochrane Database Syst Rev.

[25] Maastrup R, Hansen BM, Kronborg H, Bojesen SN, Hallum K, Frandsen A (2014). Breastfeeding progression in preterm infants is influenced by factors in infants, mothers and clinical practice: the results of a national cohort study with high breastfeeding initiation rates. PLoS One.

[26] Smith RL, Lucas R (2016). Evaluation of nursing knowledge of early initiation of breastfeeding in preterm infants in a hospital setting. J Neonatal Nurs.

[27] Theurich MA, McCool-Myers M, Koletzko B (2021). Supporting breastfeeding of small, sick and preterm neonates. Semin Perinatol.

[28] Jones E, Spencer SA (2007). Optimising the provision of human milk for preterm infants. Arch Dis Child Fetal Neonatal Ed.

[29] Holdren S, Fair C, Lehtonen L (2019). A qualitative cross-cultural analysis of NICU care culture and infant feeding in Finland and the U.S.. BMC Pregnancy Childbirth.

[30] Bishara R, Dunn MS, Merko SE, Darling P (2009). Volume of foremilk, hindmilk, and total milk produced by mothers of very preterm infants born at less than 28 weeks of gestation. J Hum Lact.

[31] Prime DK, Garbin CP, Hartmann PE, Kent JC (2012). Simultaneous breast expression in breastfeeding women is more efficacious than sequential breast expression. Breastfeed Med.

[32] Smith MM, Durkin M, Hinton VJ, Bellinger D, Kuhn L (2003). Initiation of breastfeeding among mothers of very low birth weight infants. Pediatrics.

[33] Merton RK (1995). The Thomas theorem and the matthew effect. Soc Forces.

[34] Gomez-Pomar E, Blubaugh R (2018). The baby friendly hospital initiative and the ten steps for successful breastfeeding. a critical review of the literature. J Perinatol..

[35] Bellù R, Condò M (2017). Breastfeeding promotion: evidence and problems. Pediatr Med Chir.

[36] Klotz D, Jansen S, Glanzmann R, Haiden N, Fuchs H, Gebauer C (2020). Donor human milk programs in German, Austrian and Swiss neonatal units - findings from an international survey. BMC Pediatr.

